# Antimicrobial and Cytotoxic Activity of *Cuminum Cyminum* as an Intracanal Medicament Compared to Chlorhexidine Gel 

**DOI:** 10.7508/iej.2016.01.009

**Published:** 2015-12-24

**Authors:** Abbas Abbaszadegan, Ahmad Gholami, Yasamin Ghahramani, Razieh Ghareghan, Marzieh Ghareghan, Aboozar Kazemi, Aida Iraji, Younes Ghasemi

**Affiliations:** a * Department of Endodontics, Dental School, Shiraz University of Medical Sciences, Shiraz, Iran; *; b * Pharmaceutical Sciences Research Center, Shiraz University of Medical Sciences, Shiraz, Iran; *; c *Students' Research Committee, Dental School, Shiraz University of Medical Sciences, Shiraz, Iran;*; d *Central Laboratory, Shiraz University of Medical Science, Shiraz, Iran*

**Keywords:** Antibacterial Activity, Chlorhexidine, Co-trimoxazole, *Cuminum Cyminum*, Cytotoxicity, Intracanal Medicament

## Abstract

**Introduction:**

The aims of this study were *i)* to define the chemical constituents of *Cuminum cyminum* (cumin) essential oil, *ii)* to compare the antimicrobial activity of this oil to that of chlorhexidine (CHX) and co-trimoxazole on planktonic and biofilm forms of bacteria isolated from the teeth with persistent endodontic infection and *iii)* to compare the cytotoxicity of these medicaments on L929 fibroblasts.

**Methods and Materials:**

Three groups of microorganisms [aerobic bacterial mixture, anaerobic bacterial mixture and *Enterococcus faecalis *(*E .faecalis*)] were isolated from the teeth with persistent apical periodontitis. Zone of inhibition (ZOI), minimum inhibitory concentration (MIC), minimum bactericidal concentration (MBC), minimum biofilm inhibitory concentration (MBIC) and time-kill tests were performed to assess the antimicrobial efficacy of the medicaments. Further, a cytocompatibility analysis of the medicaments was performed on L929 fibroblasts. The results obtained from disc diffusion test and mean cell viability values of the experimental medicaments were analyzed using two-way and one-way analysis of variance (ANOVA).

**Results:**

Seventeen constituents were recognized in cumin oil (predominantly cumin aldehyde and *γ*-terpinene). Co-trimoxazole showed the greatest ZOI followed by cumin and CHX. The smallest MIC and MBC belonged to co-trimoxazole followed by cumin and CHX for all groups of bacteria except for *E. faecalis* for which the MBC of cumin was smaller than co-trimoxazole. The results of time-kill assay revealed that all medicaments totally inhibited the bacterial growth in all groups after 24 h. CHX was the most cytotoxic solution while there were no significant differences between the cytocompatibility of different concentrations of cumin essential oil and co-trimoxazole.

**Conclusion:**

Cumin exhibited a strong antimicrobial efficiency against the microbial flora of the teeth with failed endodontic treatments and it was biocompatible for L929 mouse fibroblasts.

## Introduction

Endodontic infections have polymicrobial nature which usually consist of aerobic and anaerobic microbiota. *Enterococcus faecalis *(*E. faecalis*) is a facultative anaerobe, gram-positive cocci regarded as one of the most resistant microorganisms that survives after root canal treatments and plays an important role in persistent periradicular lesions [1]. Microorganisms that causes persistent apical periodontitis usually exhibit a high level of resistant to disinfection and are able to survive against harsh conditions of root canal system and organize a mature biofilm [2, 3]. Therefore, they may not be easily eliminated from the root canal space by common irrigation solutions or intracanal medicaments [4]. It may be stated that efforts to eliminate these bacteria may equate with achieving successful disinfection.

Calcium hydroxide and chlorhexidine (CHX) gel are the two most widely used intracanal medicaments. However, none of them are able to completely eradicate resistant microorganisms [5, 6]. Calcium hydroxide can hardly penetrate into the dentinal tubules and does not have sufficient effectiveness against all species of endodontic pathogens and their endotoxins [7, 8]. Furthermore, CHX is toxic to vital tissues and its toxicity is proportional to its concentration [9].

Considering the increasing bacterial resistance to antibiotics and shortcomings of calcium hydroxide and CHX as intracanal medicaments, it seems reasonable to look for other alternatives [10]. Recent researches focused on herbal alternatives to defeat resistant microorganisms harboring in the root canal system. The antimicrobial activity of some herbs such as *Arctium lappa *[11], *Morinda citrifolia* [12], *Satureja khuzistanica Jamzad* [13], *triphala, green tea polyphenols* [10], *liquorice* [14], *Syzygium aromaticum, Ocimum sanctum*, and *Cinnamomum zeylanicum* [15], *Aloe vera, Zataria multiflora* [16], *Myrtus communis* [17] and *Ferula gummosa* [18] has been evaluated against *E. faecalis* in contemporary endodontic studies.

*Cuminum cyminum *also known as cumin or Jeera (in Persian zeera) is another medicinal herb native to Mediterranean region belonging to the *Apiaceae *family. Its fruit, known as cumin seed is yellow to gray and has long oval shape with nine projections [19]. It has excellent antioxidant [20, 21], antibacterial [22, 23], antifungal [24, 25] and analgesic properties [26, 27].

The purpose of this comprehensive *in vitro *study was to determine the chemical composition of cumin essential oil and to assess its potential to be used as a root canal medicament by evaluating the cytotoxicity and antibacterial activity of this oil compared to CHX gel on aerobic bacterial mixture, anaerobic bacterial mixture and *E .faecalis *using zone of inhibition (ZOI), minimum inhibitory concentration (MIC), minimum bactericidal concentration (MBC), minimum biofilm inhibitory concentration (MBIC) and time-kill tests.

## Materials and Methods

***Isolation of microorganisms***

Three groups of microorganisms consisted of aerobic bacterial mixture, anaerobic bacterial mixture and *E. faecalis *were isolated from the teeth with persistent apical periodontitis that were candidate for root canal retreatment. Aerobic and anaerobic bacteria were not identified individually and only the mixtures were tested. However, *E. faecalis* (AGH 011) was identified by gram staining, catalase reaction, colony morphology and by the pattern of carbohydrate fermentation and enzyme production and 16s rRNA sequencing (Genebank accession No.: KF701470). 

For bacterial isolation, teeth were isolated with rubber dam and the surrounding area was disinfected with 30% hydrogen peroxide and 2.5% sodium hypochlorite for 30 sec. Then, 5% sodium thiosulphate was used to neutralize the disinfectant. The access cavity was prepared and the root filling materials were removed using Gates-Glidden drills and K-files (Dentsply Maillefer, Ballaigues, Switzerland) without employing solvents. After determination of the working length, sterile paper points were placed 1 mm short of the working length for 30 sec. Then the paper points were transferred to the sterile vials containing BHI (brain-heart infusion) broth (BHI, Merck, Darmstadt, Germany) and dispersed within vortex for 1 min. Ten-fold serial dilutions were prepared via BHI broth and 100 µL of the suspension was cultured on enriched BHI agar plates in separate aerobic and anaerobic conditions. The anaerobic condition was established by an anaerobic box (Anoxomat: MART Microbiology B.V. the Netherlands), 0% O_2_, 10% H_2_, 10% CO_2_ and 80% N_2_.

***Preparation of the medicament***

The seeds of cumin were collected from Joupar region in Kerman province, Iran and were identified and authenticated by an expert plant taxonomist, according to the morphological description and previously collected known samples. Then 200 g of the grounded seeds (powder) was mixed with 800 mL of distilled water. The essential oil was isolated using a Clevenger-type apparatus. The oil was stored in -70^º^C in order to remove the water-soluble components. In order to prepare the known concentration for a water-soluble essential oil, we initially weighted a defined concentration of the oil and dissolved it in a defined volume of dimethyl sulfoxide (DMSO). Further, the resultant solution was dissolved in a defined volume of water. The final concentration of the oil was 700 µg/mL. Gas chromatography-mass spectrophotometry (GC-MS) analysis was performed to determine the chemical composition of the cumin oil using Agilent 7000 mass spectrometer coupled to Agilent 7890A series gas chromatograph (Agilent technologies, Santa Clara, CA, USA). The constituents were identified by comparison of Kováts retention indices (KI), referring to compounds known from literature database and also by comparing their mass spectra with the Wiley library or with the published mass spectra. Relative percentage amounts were calculated from the total area under the peaks by the software of the apparatus.

CHX gel (2% gel, 20000 µg/mL, Natufarma, Passo Fundo, RS, Brazil) as another experimental group and trimethoprim-sulfamethoxazole (co-trimoxazole 400/80 µg/mL, Caspian Tamin Pharmaceutical Co; Rasht, Iran) as a control group were also used. It is notable that, as a trial, initially eight different antibiotics were tested (penicillin, gentamycin, erythromycin, ciprofloxacin, chloramphenicol, cefixime, trimethoprim and co-trimoxazole) to determine a suitable control group and selected co-trimoxazole among them because it was the most efficient antibiotic against all three groups of microorganisms tested.

***Disc diffusion method***

Disc diffusion method was used for the primary evaluation of antimicrobial susceptibility of the medicaments. Sterile water was used as a negative control group; 100 µL of each bacterial suspension (1.5×10^8^ CFU/mL) were streaked on BHI agar plates separately. After 24 h of incubation, 50 µL of each medicament was added in sterile blank 6-mm filter paper discs and placed on the plates. The plates were left under laminar air flow for 15 min and incubated for 24 h. After incubation, the zone of inhibition around each disc was measured. These procedures were repeated for 6 times.

The results obtained from disc diffusion test were analyzed using two-way analysis of variance (ANOVA). Due to the presence of interaction effect, multiple comparisons were performed using One-way ANOVA/Tamhane post hoc tests.

***Determination of MIC and MBC***

The minimum inhibitory concentration (MIC) and minimum bactericidal concentration (MBC) of cumin oil, CHX gel and co-trimoxazole against each group of microorganisms were determined via 2-fold dilution method according to the protocol suggested by Clinical Laboratory and Standard Institute [28]. DMSO in the ratio of 1:2 (v/v) was used as a vehicle of the oil. Then 10 µL of each cultured microorganisms with optical density of 600 nm (0.5 McFarland) transferred to 96-well plates containing the experimental medicaments. The positive control groups included saline, BHI, microorganisms or DMSO, BHI, microorganisms while the negative control groups had normal saline and BHI or DMSO and BHI without microorganisms. The plates were incubated for 24 h at 37^º^C. Plates were shaken for 60 sec and read at 600 nm using microplate reader spectrophotometer (BioTek, Winooski, VT, USA).

To determine MBC, the concentrations of each medicament that was equal and higher than MIC, were spread on BHI agar plates and incubated for 24 h at 37^º^C. Afterwards, the colonies were counted. The MBC was defied as the lowest concentration in which no bacterial growth was detectable on the plates. These procedures were repeated for 3 times.

**Table 1:** Chemical composition of *Cuminum cyminum*

**Component**	**Percent**
Cuminaldehyde	23.18
*γ*-Terpinen	20.47
*α*-Terpinen-7-al	17.35
*γ*-Terpinen-7-al	13.31
*β*-Pinene	12.47
*p*-cymene	6.17
1,3-Cyclohexadiene-1-methanol , 4-(1-methylethyl)	1.79
Myrcene	0.96
Sabinene	0.85
*β*-Phellandrene	0.85
*α*-Pinene	0.76
a-Phellandrene	0.47
Mentha-1,4-dien-7-ol-p	0.42
3,6-Octadien-1-ol, 3,7-dimethyl-, (Z)-	0.38
*α*-Thujene	0.29
(E)-*β*-Farnesene	0.28

***Determination of MBIC***

The minimum biofilm inhibitory concentration (MBIC) of cumin, CHX and co-trimoxazole against all groups of microorganisms were determined using a 2-fold dilution method. MBIC was known as the lowest concentration of an antimicrobial agent that inhibited the visible biofilm formation of the tested microorganisms. Briefly, microorganisms in BHI were treated with a series of 2-fold dilutions of each medicament in 96-well plates. After 24 h, 7 and 14 days of incubation, the plates were washed 2 times with saline and fixed with 10% formaldehyde and again washed twice with saline. The biofilms were stained with 0.5% crystal violet for 30 min. To eliminate crystal violet from the biofilms, the plates were treated with 200 µL of 2-propanol for 1 h, then read at 490 nm by using a plate reader spectrophotometer (VITA Easyshade; VITA Zahnfabrik, Bäd Sackingen, Germany). The absorbance of crystal violet was regarded as a real indicator for the presence of bacterial biofilm. Therefore, higher absorbance represented higher biofilm mass [29]. These procedures were repeated for 3 times.

***In vitro killing assay***

The overnight grown culture of the microorganisms in each group were centrifuged at 2500 rpm for 10 min and washed with phosphate buffer saline (PBS) and suspended in BHI medium. The prepared concentrations of the medicaments and a control group (sterile water) were incubated with an adjusted amount of the bacteria equal to a 0.5 McFarland standard in BHI medium in 96-well round bottom plates. Bacteria were harvested at 0, 1, 4 and 24 h after inoculating. Two-fold serial dilutions were made for seven times. Then, 10 μL of each dilution was spread on BHI agar plates. After an overnight incubation, the number of colony forming units (CFUs) was counted. These procedures were done in triplicate.

***Cytotoxicity on L929 fibroblasts***

To evaluate the cytotoxicity of the medicaments MTT colorimetric assay was used on L929 mouse fibroblasts. The experimental groups were 2% CHX gel, cumin essential oil at 700, 350, 175 µg/mL and co-trimoxazole at 400/80 µg/mL. Culture medium and H_2_O_2 _were used as the negative and positive controls respectively. L929 mouse fibroblasts in RPMI media (10^4^ cells) were poured into a 96-well plate and incubated for 24 h at 37^°^C in humidified atmosphere of 5% CO_2_, 95% air to reach confluence. 100 µL of each medicament which were incubated in RPMI media for 24 h at 37^°^C were poured into each well. After an overnight of incubation, the medium was removed and the wells were washed by 25 µL of MTT [3-(4, 5 Dimethylthiazol-2- yl)-2, 5-diphenyl tetrazolium bromide] (Sigma-Aldrich Co., St. Louis, MO, USA) stock solution and incubated again for 4 h. Afterwards, 100 µL of DMSO was added to each well to dissolve the formazan crystals and subsequently the absorption of the solutions were read via an ELISA plate reader (PowerWaveTM X52, BioTek Instruments Inc., Potton, UK) at a wavelength of 540 nm. The cell viability was considered as the percentage of mean optical density values of each medicament compared with the optical density value of the negative control. These procedures were repeated for 3 times. One-way ANOVA and Tamhane post hoc tests were used to evaluate the differences in the mean cell viability values of the experimental medicaments. All analyses were performed using SPSS 15.0 software (Statistical Package for Social Science, SPSS, version 15.0, SPSS, Chicago, IL, USA).

## Results

***Chemical composition of the cumin essential oil***

The results obtained by GC-MS analysis of the essential oil of cumin are presented in Table 1. Seventeen constituents were identified representing 100 % of the total oil. Cumin aldehyde had the highest proportion (23%) followed by *γ*-terpinen (20%), *α*-terpinen-7-al (17%), *γ*-terpinen-7-al (13%), *β*-pinene (12%) and *p-*cymene (6%).

***Antimicrobial activity***

**Figure 1:**
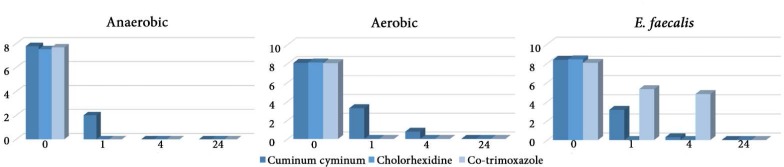
Time killing assay for each medicament in different time intervals (h). Vertical bar shows number of viable bacteria

Table 2 shows the results of disc diffusion, MIC and MBC of the medicaments for each group of microorganism. Sterile water did not induce an inhibition zone. Co-trimoxazole showed the greatest ZOI followed by the cumin and CHX. The ZOI induced by cumin and co-trimoxazole were not statistically different for all three groups of bacteria while this difference was significant for CHX. DMSO showed no MIC and MBC. The smallest MIC and MBC was for co-trimoxazole followed by cumin and CHX for all groups of bacteria except for *E. faecalis* for which the MBC of cumin was smaller than co-trimoxazole. This trend was also similar for MBIC results in which the smallest MBIC against all groups of bacteria in all time intervals belonged to co-trimoxazole followed by cumin and CHX. The results of MBIC are summarized in Table 3. The results of time-kill assay revealed that all medicaments totally inhibited the growth of planktonic bacteria in all groups after 24 h. However, the killing efficacy of the tested medicaments was time-dependent. The results are shown in Figure 1.

**Table 2:** Mean (SD) of antibacterial activity of cumin, chlorhexidine and co-trimoxazole. Different letters show the significant difference

	**Aerobic**	**Anaerobic**	***E. faecalis***
Disc diffusion in mm*			
***Cuminum cyminum***	30.76 ( 5.3) ^a^	30.5 (9.43)^ a^	28.75 (13.74^ )ab^
**Chlorhexidine**	14.17 ( 0.26) ^b^	13.22 (1.64)^ b^	18.75 (2.49)^ a^
**Co-trimoxazole**	31 (2.61)^ a^	42 (4.24)^ a^	41 (8.67^ )b^
**MIC in µg/mL **			
***Cuminum cyminum***	89.37(51.88)	14.29 (14.86)	185.91 (142.31)
**Chlorhexidine**	2083.33(645.5)	1485.33 (510.31)	2708.33 (1228.99)
**Co-trimoxazole**	0.09(0.03)	0.09 (0.03)	93.73 (63.15)
**MBC in µg/mL**			
***Cuminum cyminum***	145 (0)	149 (0)	175 (0)
**Chlorhexidine**	2500 (0)	1250 (0)	2080 (0)
**Co-trimoxazole**	100 (0)	100 (0)	400 (0)

**Table 3:** Mean (SD) of minimum biofilm inhibitory concentration (in µg/mL) of the medicaments in different time intervals

**Aerobic**	**1 day**	**7 days**	**14 days**
***Cuminum cyminum***	291.67 (101.04)	50.98 (33.49)	18.36 (22.67)
**Chlorhexidine**	8333.33 (2886.75)	1666.67 (721.69)	543.33 (625.65)
**Co-trimoxazole**	16.6 (7.24)	2 (0.69)	0.83 (0.65)
**Anaerobic**			
***Cuminum cyminum***	87.5 (0)	21.7 (0)	4.2 (0)
**Chlorhexidine**	5000 (0)	1666.67 (721.69)	22.67 (32.33)
**Co-trimoxazole**	7.07 (5.03)	1.2(0)	1.2 (0)
***E. faecalis***			
***Cuminum cyminum***	291.67 (101.04)	175(0)	1.79 (1.13)
**Chlorhexidine**	5000 (0)	4200(1385.64)	41.33 (32.33)
**Co-trimoxazole**	166.67 (57.74)	166.67(57.74)	1.63 (1.34)

***Antimicrobial activity***

Table 2 shows the results of disc diffusion, MIC and MBC of the medicaments for each group of microorganism. Sterile water did not induce an inhibition zone. Co-trimoxazole showed the greatest ZOI followed by the cumin and CHX. The ZOI induced by cumin and co-trimoxazole were not statistically different for all three groups of bacteria while this difference was significant for CHX. DMSO showed no MIC and MBC. The smallest MIC and MBC was for co-trimoxazole followed by cumin and CHX for all groups of bacteria except for *E. faecalis* for which the MBC of cumin was smaller than co-trimoxazole. This trend was also similar for MBIC results in which the smallest MBIC against all groups of bacteria in all time intervals belonged to co-trimoxazole followed by cumin and CHX. The results of MBIC are summarized in Table 3. The results of time-kill assay revealed that all medicaments totally inhibited the growth of planktonic bacteria in all groups after 24 h. However, the killing efficacy of the tested medicaments was time-dependent. The results are shown in Figure 1. 

***Cytotoxicity assessment***

**Figure 2:**
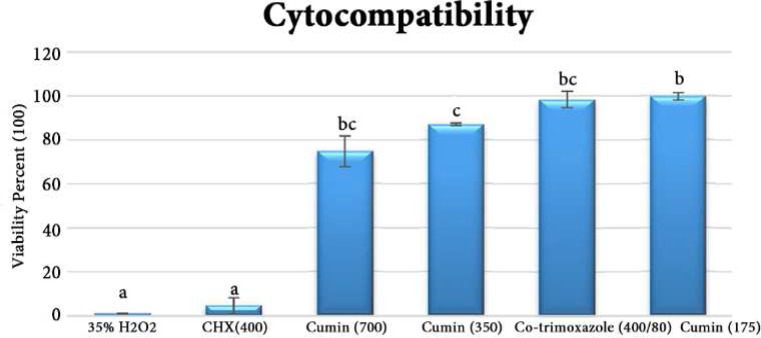
Mean viability (%) of cells after 24 h contact with test materials (concentration in µg/mL). Equal letters show lack of significant difference (*P*>0.05

Figure 2 shows the mean cell viability (%) of L929 fibroblasts in each experimental group. One-way ANOVA/Tamhane tests showed a statistically significant difference between the groups. Also 35% H_2_O_2_ as positive control caused 98.9% cell death. There was no statistically significant difference between the cytotoxicity of the 2% CHX and 35% H_2_O_2_. There were no significant differences between the cytocompatibility of different concentrations of cumin essential oil and co-trimoxazole at its full concentration on L929 fibroblasts. 

## Discussion

The current research was performed to investigate the potential of using cumin essential oil as an intracanal medicament. The chemical components of the cumin essential oil were determined to avoid the lack of standardization and also to have a better understanding of its bioactivity.

The use of antimicrobial intracanal medicaments with reasonable biocompatibility will result in elimination of the remained organisms in root canal system and increase the rate of favorable outcomes of root canal treatment. Plant essential oils are potential sources of new antimicrobial compounds especially against bacterial pathogens. These essential oils have hydrophobicity characteristics. Therefore, they can degrade the lipids of the bacterial cell wall and the mitochondria and subsequently destroy the bacterial structures [30].

The composition of herbal essential oils can be variable depending on several factors such as geographic region, harvest time, extraction method and the type of culture. However, this diversity in composition can be regarded as a pharmaceutical opportunity in a way that different therapeutic usage of the same plant species grown in different regions become possible. In the current study, the GC-MS analysis of the cumin essential oil revealed 17 constituents in its composition with the predomination of cumin aldehyde and *γ*-terpinen, *α*-terpinen, *β*-pinene and *p*-cymen. This finding was consistent with the previous studies [19, 22, 31]. However, the sequences of the major components were different in literature [32, 33].

It has been demonstrated that the aldehyde and ketone components of the essential oils relate to their level of antimicrobial activity [34]. Furthermore, different researches revealed that the presence of aldehyde, terpinen, pinene and cymen are responsible for many biological effects [35]. In line with these findings, major components of the tested essential oil were similar to them.

In the second part of this study, the antibacterial performance of the cumin essential oil was assessed against the bacterial flora of the teeth with failed root canal treatment. Therefore, in this study, three groups of bacteria consisted of aerobic and anaerobic microorganisms and a strain of *E. faecalis* were isolated from the previously root filled teeth with failed endodontic treatment and this medicinal plant was tested on them. The reason to select this plant was its strong background in history regarding the antimicrobial activity. Previous researches had shown that cumin has promising antibacterial, antifungal and antioxidant activity [20, 21, 23]. The reasonable antibacterial activity of the cumin essential oil has been proven against some gram-positive and gram-negative bacteria including *E. faecalis *[22] and some species of yeasts including *Candida albicans *[22, 36]. 

In the current research, disc diffusion, MIC, MBC and MBIC value of this essential oil were determined and the time-kill assay was performed for better understanding of its effects in different time intervals. The results were compared with that of CHX gel since it was one of the most famous intracanal medicaments. Co-trimoxazole was also used for all comparisons as a positive control group according to the pre-trial results obtained in this study. We found that cumin essential oil was a more potent antimicrobial agent compared to CHX against all groups of microorganisms. This high level of antimicrobial activity can be attributed to the presence of cumin aldehyde and other major components in the composition of the essential oil.

The results of time-kill assay demonstrated the slow-acting nature of cumin essential oil compared to CHX since the antimicrobial activity of this oil enhanced with the increase in contact time. This finding was consistent with the results of pervious researches which revealed that a prolonged contact time is required for herbal remedies [10, 16, 37].

In the current study we found that cumin was effective in elimination of the tested microorganisms in the MIC and MBC range of 14-185 µg/mL. Partially in line with our findings, a previous research on Tunisian cumin reported that the MIC of cumin essential oil were about 78-150 µg/mL against a panel of gram positive and gram negative microorganisms including *E. faecalis *[22].

Another advantage of using herbal medicaments comparing to their synthetic counterparts is their lower toxicity [18]. To the best of our knowledge there is no study on cytotoxicity of cumin on the normal cell-lines. In the present study, an immortalized L929 cell line was employed to compare the cytotoxicity of the experimental medicaments. This cell line was chosen because it is a well-characterized cell model and has been previously used to assess the cytotoxic effects of dental materials [38]. Our results showed that there was no statistically significant difference between the cytotoxicity of 2% CHX and 35% H_2_O_2_. Moreover, cumin at any concentration had significantly lower toxicity compared to 2% CHX. This high level of CHX toxicity has also been demonstrated previously [9, 38].

Although the results of the current study support that the cumin essential oil could be an effective and biocompatible antibacterial agent, it is noteworthy that possible interactions between chemical, physical and pharmacological properties of this oil with dentinal tubules or root canal filling materials are still unclear. Furthermore, the level of hydrophobicity of this plant essential oils and their relative possible effect on effectiveness of the antimicrobial action is another issue remained to be investigated. Therefore, a conclusive comment on its application in root canal treatment should not be made until further evaluations have been performed on animal or human models.

## Conclusion

Within the limitations of the present study it was concluded that *Cuminum cyminum* essential oil exhibited a strong antimicrobial activity against the microbial flora of the teeth with failed endodontic treatments and it was biocompatible for L929 mouse fibroblasts.
